# Development of *Clostridium difficile* R20291ΔPaLoc model strains and *in vitro* methodologies reveals CdtR is required for the production of CDT to cytotoxic levels

**DOI:** 10.1016/j.anaerobe.2017.01.009

**Published:** 2017-04

**Authors:** T.W. Bilverstone, N.L. Kinsmore, N.P. Minton, S.A. Kuehne

**Affiliations:** a Clostridia Research Group, BBSRC/EPSRC Synthetic Biology Research Centre (SBRC), School of Life Sciences, Centre for Biomolecular Sciences, The University of Nottingham, Nottingham, NG7 2RD, UK; b NIHR Nottingham Digestive Diseases, (NDCC) Biomedical Research Unit, Nottingham University Hospitals NHS Trust and the University of Nottingham, NG7 2RD, UK

**Keywords:** *C. difficile*, PaLoc, Binary toxin, CdtR, Virulence

## Abstract

Assessing the regulation of *Clostridium difficile* transferase (CDT), is complicated by the presence of a Pathogenicity locus (PaLoc) which encodes Toxins A and B. Here we developed R20291ΔPaLoc model strains and cell-based assays to quantify CDT-mediated virulence. Their application demonstrated that the transcriptional regulator, CdtR, was required for CDT-mediated cytotoxicity.

## Highlights

• Model strains to study the *C. difficile* binary toxin, CDT, were generated by deleting the PaLoc in R20291.
• The gene encoding CdtR was deleted and re-integrated on the chromosome in the model strains.
• *In vitro* Cytotoxicity assays were established to assess CDT-mediated virulence.
• CdtR was required for the production of CDT to cytotoxic levels towards Vero cells.

*Clostridium difficile* infection (CDI) is the leading cause of hospital associated diarrhoea in the developed world. In 2011, there were an estimated 453,000 cases and 29,000 deaths in the USA alone [1]. The main virulence factors of *C. difficile* are Toxin A (TcdA) and Toxin B (TcdB) whose genes reside on a 19.6 Kb pathogenicity locus (PaLoc) [2]. Some hypervirulent strains responsible for outbreaks and severe cases of disease, in particular BI/NAP1/027 strains including the archetypal strain R20291, produce an additional toxin, the binary toxin or *Clostridium difficile* transferase (CDT) [3]. Owing to the overwhelming potency of TcdA and TcdB, it is difficult to study the genetic regulation of CDT using cell-based assays or *in vivo* approaches. Moreover, the main approach for accurately quantifying CDT is through ADP-ribosyltransferase assays which utilise radioactive phosphorus 32 and require adherence to stringent safety precautions, although, a prototypal ELISA assay reliant on an antibody against the B subunit of the *Clostridium perfringens* Iota Toxin, has also been described [4]. In light of these impediments, the regulation of CDT remains relatively uncharacterised. However, a gene encoding the transcriptional regulator CdtR belonging to the LytTR family, was discovered upstream of *cdtA* and *cdtB* (for locus arrangement see Fig. S1b), and was shown to be required for the maximal expression of CDT [5]. By introducing CDT on autonomous plasmids to strains which lack the toxin, with or without the *cdtR* gene, the presence of *cdtR* was shown to increase CDT production by 17-fold [5].

The development of model strains devoid of TcdA and TcdB activity, coupled with reliable *in vitro* cell-based assays for the quantification of CDT, would facilitate the study of CDT regulation. We used allelic exchange technology, to delete the entire protein-coding region of the PaLoc in the PCR-Ribotype 027 strain R20291 in which the *pyrE* gene had been deleted to facilitate genome engineering [6]. Deletion of the PaLoc has not been previously described and represents the largest deletion of gDNA in *C. difficile* reported to date. We achieved this by the construction of a knockout cassette homologous to the regions up/downstream of the PaLoc. The cassette was conjugated into *C. difficile* from *E. coli* CA434 and gene deletion was achieved through two homologous recombination events [7]. In addition, we used the same methodology to make the first reported in-frame deletion mutant for *cdtR*. The mutant created was designated R20291Δ*pyrE*Δ*cdtR*. We also made triple mutants in which *pyrE*, *cdtR* and the PaLoc had all been deleted. Gene deletions were authenticated by PCR of the target regions (see supplementary material) using the appropriate primers (Table S2). Following the authentication of the deletion mutants, the *pyrE* allele was repaired in strains R20291Δ*pyrE*ΔPaLoc and R20291Δ*pyrE*ΔPaLocΔ*cdtR* using the plasmid pMTL-YN2 [6]. In parallel, *cdtR* along with the 273bp upstream region encompassing its native promoter, was cloned into pMTL-YN2C [6], which was used to simultaneously repair *pyrE,* and integrate the promoter-*cdtR* construct into the genome at the *pyrE* locus to generate the complemented strain R20291ΔPaLocΔcdtR*cdtR.

**Fig. 1:**
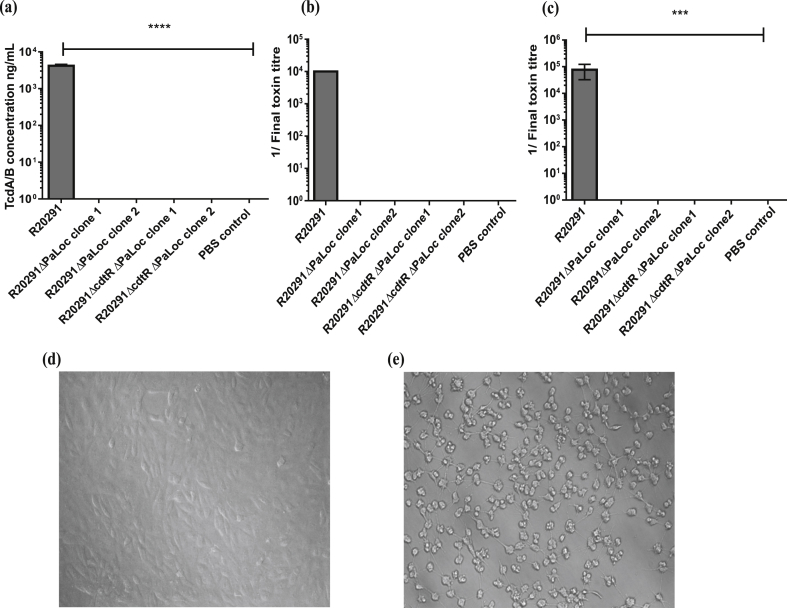
(a) Concentration of combined TcdA and TcdB detected in the supernatants of the ΔPaLoc model strains and wild-type controls, as assessed by ELISA. Data represent the mean ± SD of ≥4 replicate values. P=<0.0001 as determined by one-way ANOVA followed by Dunnett's multiple comparison test. The R^2^ value determined by assaying known concentrations of combined TcdA and TcdB from 0 to 125 ng/ml was 0.9955 and the following equation was used to convert absorbance values to toxin concentrations (x-0.1015)/0.0205 after accounting for the initial dilutions. (b) Cytotoxicity assay using Vero cells treated with 24 h supernatants from the ΔPaLoc model strains and wild-type controls. The end point represents the greatest dilution at which >50% of the cells had rounded. Data represent the mean ± SD of 5 replicate values. (c) Cytotoxicity assay using Vero cells treated with 48 h supernatants from the ΔPaLoc model strains and wild-type controls. Data represent the mean ± SD of ≥4 replicate values. P = ***<0.0002, ****<0.0001 as determined by one-way ANOVA followed by Dunnett's multiple comparison test. (d) Representative image of a Vero cell monolayer treated with a 1 × 10-1 dilution of R20291ΔPaLoc supernatant. (e) Representative image of a Vero cell monolayer treated with a 1 × 10-1 dilution of R20291 supernatant.

To validate the TcdA/B minus phenotype of the constructed model strains, the combined concentration of TcdA and TcdB was quantified in 48 h supernatants from ≥4 replicate cultures per strain, by ELISA. No toxins could be detected in the supernatant of any of the tested clones and they were indistinguishable from the PBS controls (Fig. 1a). The parental strain R20291 secreted approximately 4200 ng/ml combined TcdA and TcdB. In addition, the absence of TcdA and TcdB was confirmed through cytotoxicity assays using Vero cells (kidney cells from the African green monkey) with 24 h and 48 h supernatants. At the 24 h and 48 h time point, the R20291 supernatant rounded >50% of the cells down to a 1 × 10^−4^ and 1 × 10^−5^ dilution, respectively (Fig. 1b and c). In contrast, supernatants derived from the model strains in which the PaLoc had been deleted, caused no cell rounding at any of the dilutions (1 × 10^−1^ – 1 × 10^−8^) tested (Fig. 1b and c) at the same time points. Fig. 1d and e, are representative images of Vero cells treated with a 10^−1^ dilution of the supernatants of strains R20291ΔPaLoc and R20291, respectively. A confluent monolayer of healthy cells is visible in the former image whereas all of the cells had clearly rounded in the latter image.

**Fig. 2:**
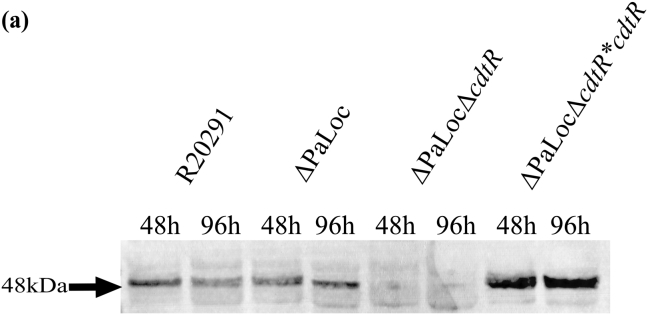
Western blot of 48 and 96 h supernatants detected with an anti-CdtA:HRP antibody derived from strains R20291, R20291ΔPaLoc, R20291Δ*cdtR*ΔPaLoc and R20291Δ*cdtR*ΔPaLoc*cdtR.

Following validation of the TcdA/B minus phenotype, we investigated CDT production and tested the effects of *cdtR* deletion. Our initial assessments relied on Western blot procedures using an antibody against the enzymatic subunit of CDT, CdtA. Supernatants were collected and processed at 48 h and 96 h time points from R20291, R20291ΔPaLoc and R20291ΔPaLocΔ*cdtR*. Distinct 48 kDa bands were detectable across both time points for R20291 and R20291ΔPaLoc (Fig. 2). However, no distinct bands were detected from the supernatants of strain R20291ΔPaLocΔ*cdtR*. This indicated that without functional CdtR, CdtA production was either completely ablated, or its production was reduced to concentrations below the detection threshold of the antibody. Deletion of the PaLoc appeared to have no discernible effect on CDT production. Complementation of *cdtR* at the *pyrE* locus restored the production of CdtA (Fig. 2). In fact, the R20291ΔPaLocΔcdtR*cdtR strain appeared to overexpress the phenotype. An insertional mutant has recently been generated for *cdtR*, and in that study, residual CdtA was clearly detectable by Western blot following gene interruption [8]. However, owing to the nature of group II intron insertional mutagenesis, the residual expression may be a polar effect from the promoter of the erythromycin resistance marker since the authors describe an antisense insertion [8].

The glucosylation of Rho family GTPAses by TcdA and TcdB, leads to cytoskeletal disorganisation and consequently cell rounding [9]. This forms the basis of cytotoxicity assays for the quantification of TcdA and TcdB. CdtA, ADP-ribosylates monomeric actin, leading to actin depolymerisation and consequently cell rounding [10]. With the masking effects of TcdA and TcdB removed, it should now be possible to measure the cytotoxic effects of CDT in model strain-derived supernatants. Before doing so, the CdtB binding subunit of CDT requires activation by proteolytic cleavage. Without which, CdtA cannot be taken up into mammalian cells [11], [12]. An effective strategy for achieving this was established by the treatment of supernatants with 400 μg/ml trypsin and its subsequent inhibition with 200 μg/ml trypsin inhibitor (see Supplementary Material). Following CDT activation, model strain-derived supernatants were diluted in a 4-fold series and were applied to monolayers of Vero cells.

**Fig. 3:**
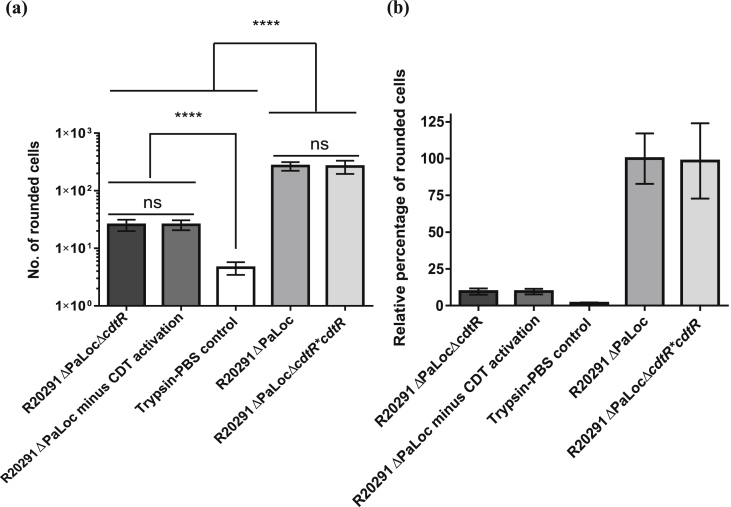
(a) Total number of rounded cells (b) Percentage of rounded cells relative to complete virulence by strain R20291ΔPaLoc, for Vero cells treated with the model strains and relative controls. Data represent the mean ± SD of five replicate values P =<0.0001 as determined by one-way ANOVA followed by Dunnett's multiple comparison test.

To determine the cytotoxicity of the supernatants, the total number of rounded cells was determined from images of cells treated with undiluted supernatants of each replicate culture per strain (n = 5). Since the well-plate was seeded with the same density of Vero cell suspension, and each replicate bacterial culture was normalised to the same OD (0.135), there shouldn't be any major difference in the total number of cells, or number of rounded cells between conditions, other than in response to virulence factors in the supernatant. On visualisation of the cells treated with supernatants derived from R20291ΔPaLoc, practically all of the cells had rounded (Fig. S3a–c). An average of 268 rounded cells were counted, representing the 100% rounded cell benchmark, i.e. complete cytotoxicity (Fig. 3). An average of 25.6 rounded cells were detected in the monolayers treated with the R20291ΔPaLocΔ*cdtR* supernatant (Fig. S3d–f), corresponding to only 9.6% of the cytotoxic effect of R20291ΔPaLoc (Fig. 3). This residual toxicity, however, was not CDT-mediated, since the CDT-minus control comprising R20291ΔPaLoc supernatant without the proteolytic activation of CdtB, and which consequently could not mediate cellular entry of CdtA [11], [12], also led to an average of 25.6 rounded cells (Fig. S3j−l), thus demonstrating the involvement of other non-toxin virulence factors. Owing to variation between samples, this represented 9.54% of the relative cytotoxicity of the R20291ΔPaLoc supernatant (Fig. 3). Supernatants derived from the complemented strain R20291ΔPaLocΔcdtR*cdtR rounded an average of 264 cells (Fig S3m−o), which is 1.6% fewer cells than observed for the R20291ΔPaLoc supernatant (Fig. 3). For the trypsin control (sterile PBS treated with trypsin and subsequently trypsin inhibitor), (Fig. S3g−i), only 4.6 cells had rounded, representing 1.7% of the relative cytotoxicity of R20291ΔPaLoc (Fig. 3). Trypsinisation and trypsin inhibition therefore, was not adversely affecting the cells. Supernatants subjected to a 1 × 4^−1^ dilution failed to round any cells compared with the trypsin control for the supernatants of strains R20291ΔPaLoc and R20291ΔPaLocΔ*cdtR*. However, the R20291ΔPaLocΔcdtR*cdtR-derived supernatant still rounded all of the cells at this dilution (Fig. S4), thus indicating that the expression of *cdtR* is at least 4-fold higher than the parental strain. These data suggest that, within the parameters tested, CdtR is required for the production of CDT to levels which are cytotoxic towards Vero cell lines, thus demonstrating the importance of this transcriptional regulator.

In summary, we have developed model strains for the study of CDT without interference from TcdA and TcdB and demonstrated their utility. We have coupled them with cytotoxicity assays using Vero cell lines, to develop a reproducible method for studying the regulation of CDT. This was achieved by comparative assessment of the CDT-mediated virulence of genetic mutants, in our case R20291ΔPaLocΔ*cdtR*, with the control strain R20291ΔPaLoc. Application of the model strains and cytotoxicity assays, confirmed the role of CdtR in CDT production. The availability of these strains will facilitate the discovery and analysis of those determinants involved not only in CDT production, but also other non-toxin, secreted virulence factors.

## Author contributions

NLK constructed strain R20291Δ*pyrE*Δ*cdtR*. TWB made all the remaining strains, conducted the experimental procedures and analysed the data. TWB, NPM and SAK conceived the project and wrote the manuscript.

## Conflicts of interest

The authors declare no conflicts of interest.

## References

[1] Lessa F.C., Mu Y., Bamberg W.M., Beldavs Z.G., Dumyati G.K., Dunn J.R., Farley M.M., Holzbauer S.M., Meek J.I., Phipps E.C., Wilson L.E., Winston L.G., Cohen J.A., Limbago B.M., Fridkin S.K., Gerding D.N., McDonald L.C. (2015). Burden of *Clostridium difficile* infection in the United States. N. Engl. J. Med..

[2] Vedantam G., Clark A., Chu M., McQuade R., Mallozzi M., Viswanathan V.K. (2012). *Clostridium difficile* infection: toxins and non-toxin virulence factors, and their contributions to disease establishment and host response. Gut Microbes.

[3] O'Connor J.R., Johnson S., Gerding D.N. (2009). *Clostridium difficile* infection caused by the epidemic BI/NAP1/027 strain. Gastroenterology.

[4] Carman R.J., Stevens A.L., Lyerly M.W., Hiltonsmith M.F., Stiles B.G., Wilkins T.D. (2011). *Clostridium difficile* binary toxin (CDT) and diarrhea. Anaerobe.

[5] Carter G.P., Lyras D., Allen D.L., Mackin K.E., Howarth P.M., O'Connor J.R., Rood J.I. (2007). Binary toxin production in *Clostridium difficile* is regulated by CdtR, a LytTR family response regulator. J. Bacteriol..

[6] Ng Y.K., Ehsaan M., Philip S., Collery M.M., Janoir C., Collignon A., Cartman S.T., Minton N.P. (2013). Expanding the repertoire of gene tools for precise manipulation of the *Clostridium difficile* genome: allelic exchange using *pyrE* alleles. PLoS One.

[7] Heap J.T., Ehsaan M., Cooksley C.M., Ng Y.K., Cartman S.T., Winzer K., Minton N.P. (2012). Integration of DNA into bacterial chromosomes from plasmids without a counter-selection marker. Nucleic Acids Res..

[8] Lyon S.A., Hutton M.L., Rood J.I., Cheung J.K., Lyras D. (2016). CdtR regulates TcdA and TcdB production in *Clostridium difficile*. PLoS Pathog..

[9] Voth D.E., Ballard J.D. (2005). *Clostridium difficile* toxins: mechanism of action and role in disease. Clin. Microbiol. Rev..

[10] Gerding D.N., Johnson S., Rupnik M., Aktories K. (2014). *Clostridium difficile* binary toxin CDT: mechanism, epidemiology, and potential clinical importance. Gut Microbes.

[11] Fernie D.S., Knights J.M., Thomson R.O., Carman R.J. (1984). Rabbit enterotoxaemia: purification and preliminary characterisation of a toxin produced by Clostridium spiroforme. FEMS Microbiol. Lett..

[12] Perelle S., Gibert M., Bourlioux P., Corthier G., Popoff M.R. (1997). Production of a complete binary toxin (actin-specific ADP-ribosyltransferase) by *Clostridium difficile* CD196. Infect. Immun..

