# Supplementary material for: Development of Clostridium difficile R20291ΔPaLoc model strains and in vitro methodologies reveals CdtR is required for the production of CDT to cytotoxic levels
Source: Anaerobe. 2017 Apr;44:51–4. doi: 10.1016/j.anaerobe.2017.01.009 (PMC5408908; doi:10.1016/j.anaerobe.2017.01.009)
Supplement: Supplementary file 1 [file mmc1.docx]

**Supplementary Information**

Development of *Clostridium difficile* R20291ΔPaLoc model strains and *in vitro* methodologies reveals CdtR is required for the production of CDT to cytotoxic levels.

Authors: T.W. Bilverstone^1^, N.L. Kinsmore^1,2^, N.P. Minton*^1,2^ and S.A. Kuehne^1,2,3^.

^1^ Clostridia Research Group, BBSRC/EPSRC Synthetic Biology Research Centre (SBRC), School of Life Sciences, Centre for Biomolecular Sciences, The University of Nottingham, Nottingham, NG7 2RD, UK.

^2^ NIHR Nottingham Digestive Diseases, (NDCC) Biomedical Research Unit, at Nottingham University Hospitals NHS Trust and the University of Nottingham NG7 2RD, UK.

^3^ Present address: Oral Microbiology Group, School of Dentistry, College of Medical and Dental Sciences, The University of Birmingham, Birmingham, B5 7EG, UK

Corresponding author: nigel.minton@nottingham.ac.uk

**Experimental procedures**

General bacterial culture

*E. coli* cultures were grown on Luria Bertani (LB) medium and incubated at 37°C. When necessary, liquid LB and solid LB were either supplemented with 25 or 50µg/ml chloramphenicol (Cm), respectively (LB Cm). Kanamycin (Km) was added at 50µg/ml for selection of the conjugal plasmid in CA434 (LB Cm Km). *C. difficile* was routinely maintained on Brain heart infusion medium (Oxoid) supplemented with 5µg/ml yeast extract, 0.1% w/v L-cysteine (BHIS), *C. difficile* selective supplement comprising 250µg/ml D-cycloserine, and 8 µg/ml cefoxitin (Oxoid) (BHIS CC), and where necessary, an additional supplementation of 15 µg/ml thiamphenicol (BHIS CCTM). *C. difficile* cultures were grown in an anaerobic workstation (Don Whitley) in an anaerobic gas mixture comprising 80% N_2_, 10% H_2_ and 10% CO_2_.

For phenotypic assays assessing virulence including toxin ELISA, cell cytotoxicity and toxin detection by Western blot, *C. difficile* strains were cultured overnight on BHIS CC plates. A total of five individual colonies were selected per strain, or per clone of each strain, and inoculated into 1ml of BHIS for 4-6h. This represents the experimental replicates. A dilution series was made from the resultant cultures in fresh BHIS in a 96 well plate and the plate incubated overnight. The following morning, 20µl of the 10^-3^ dilution of each replicate was inoculated into 20ml of sterile pre-reduced, pre-warmed tryptose-yeast extract (TY) medium. This resulted in consistent optical density OD_600_nm (OD) values of around 0.027.

Bacterial conjugation

Shuttle plasmids were conjugated from *E. coli* CA434 into *C. difficile* essentially as previously described [[1](#_ENREF_1)] but with the following adaptations: the conjugal mating was extended to 20-24h and after conjugation the mixture was plated onto four selective plates.

Strain construction

Bacterial strains and plasmids used in this study are detailed in Table S1 and oligonucleotide primers are detailed in Table S2. In-frame deletions were generated by allelic exchange as previously described [[2](#_ENREF_2)]. The knockout cassette (KOC) for the deletion of *cdtR* was generated by PCR amplification of a left homology arm corresponding to a 700bp region upstream of *cdtR* with a flanking 5’-*Sbf*I site and the amplification of a right homology arm corresponding to a 700bp region downstream of *cdtR* with a flanking 3’-*Asc*I site. The left and right homology arms were generated using primer sets *cdtR* KOC LAL/LAR and *cdtR* KOC RAL/RAR, respectively. The arms were designed with regions of homology to each other to facilitate Splicing by overlap extension PCR using the respective LAL and RAR primers. The resultant *Sbf*I -*Asc*I fragment was cloned into pMTL-YN4 (accession: JX465736) to generate the finished KOC: pMTL-YN4-cdtR KOC (Fig. S1a). The KOC was subsequently conjugated into R20291∆*pyrE* from *E. coli* CA434. Transconjugant *C. difficile* was selected and maintained on BHIS CCTM. Single-cross over (SCO) mutants in which the plasmid had inserted into the chromosome were screened using either YN4 F and *cdtR* diag R primers or *cdtR* diag F and YN4 R primers, depending on the orientation of the recombination event. SCO mutants were then grown to purity on non-selective BHIS plates, collected, and resuspended in phosphate-buffered saline (PBS). The resultant suspension was diluted to 1x10^-3^ in PBS and plated on *C. difficile* minimal media (CDMM) [[3](#_ENREF_3)], containing 500µg/ml 5-fluoroorotic acid (FOA) and 1µg/ml uracil to select for double cross-over (DCO) mutants [[4](#_ENREF_4)]. Genomic DNA was extracted from FOA-resistant colonies and DCO status was confirmed by PCR using *cdtR* diag F and *cdtR* diag R (Fig. S1c), followed by confirmation by Sanger sequencing (Source Bioscience). Stable, confirmed DCO mutants were harvested in BHIS containing 10% v/v glycerol and stored at -80°C. The primer binding sites are depicted in Fig. S1b.

A stable R20291∆*pyrE*∆*cdtR* mutant was taken forward along with R20291∆*pyrE* to construct the ∆PaLoc model strains. The procedures mimicked those described above but used primers specific to the PaLoc application. The left and right arms (933bp and 936bp) were amplified with PaLoc KOC LAL/LAR and PaLoc KOC RAL/RAR respectively, and PaLoc KOC LAL/RAR primers were used to splice the products together. The KOC was constructed (Fig. S2a), and conjugated into the respective strains as mentioned above. SCO clones were identified using YN4 F/R and PaLoc diag F/R primers and two independent SCO clones in each strain background were taken forward to generate DCO mutants. DCO mutants were confirmed by two independent PCR reactions. One reaction used primers PaLoc diag F and R to amplify across the entire deleted PaLoc region and the other used PaLoc int F and R to amplify an internal fragment of the PaLoc spanning *tcdB*, *E* and *A* (Fig. S2c). The respective binding sites for each primer set are depicted in Fig. S2b. The PCR amplicons for primer set PaLoc diag F and R were Sanger sequenced and confirmed DCO mutants were harvested in BHIS containing 10% v/v glycerol and stored at -80°C.

The *pyrE* allele was repaired in R20291∆*pyrE*∆PaLoc and R20291∆*pyrE*∆PaLoc∆*cdtR* using the ACE (Allele-Coupled Exchange) vector pMTL-YN2 as described elsewhere [[2](#_ENREF_2)]. In parallel, *cdtR* together with its native promoter (region 273bp upstream of start codon) was knocked-in at the *pyrE* locus with simultaneous repair of *pyrE* in strain R20291∆*pyrE*∆PaLoc∆*cdtR* to generate strain R20291∆PaLoc∆*cdtR***cdtR.* This was achieved by PCR amplification of the promoter-*cdtR* construct with flanking 5’-*Not*I and 3’-*Bam*HI sites using primers Promoter-cdtR F and *cdtR* R. The construct was subsequently cloned into the complementation vector pMTL-YN2C (accession: JX465733) [[2](#_ENREF_2)]. Thiamphenicol sensitive clones were grown to purity and the genotype confirmed by PCR using primers *pyrE* WT F and *cdtR* R. Amplicons were confirmed by Sanger sequencing.

ELISA quantification of Toxin A and B

Strains were grown in TY as described previously. After 48h, cultures were collected, the OD measured, and each culture normalised to the lowest OD value. Normalised samples were centrifuged and the supernatant passed through a 0.22µm filter and frozen at -20°C until required (<1 week). Samples were thawed and diluted 1X10^1^ – 1X10^8^ in sterile PBS. Diluted samples were processed for ELISA quantification of total TcdA and TcdB using a C. DIFFICILE Tox A/B II detections kit (TechLab) according to the manufacturer’s instructions. Absorbance values were converted into approximate toxin concentrations by determining the R^2^ value of the assay’s standard curve by running a range of defined combined TcdA and TcdB toxin standards from 0-125ng/ml (The Native Antigen Company).

Cell cytotoxicity assays for TcdA and TcdB

Vero cells were maintained in Dulbecco’s modified eagle medium (DMEM) (Life Technologies) containing foetal bovine serum, penicillin and streptomycin and incubated at 37°C with 5% CO_2_. Cells were cultured into 96 well-plates in 100µl volumes and incubated for 24h to form a monolayer. Toxin supernatants were collected at 24h and 48h, normalised and processed as described above to generate a 10-fold dilution series. A 50µl aliquot of each supernatant dilution per replicate was applied to the respective wells. Following overnight incubation, the cell monolayer was assessed for cell rounding under an inverted microscope. The end point (1/final toxin titre) was determined for each replicate as the greatest dilution at which at least 50% of the cells had rounded.

Cell cytotoxicity assays for CDT

*C. difficile* supernatants were collected after 96h and processed as described above. CdtB was proteolytically activated in the normalised supernatants with treatment of 400µg/ml trypsin (from bovine pancreas) (Sigma Aldrich) for 1h at room temperature. Following trypsinisation, trypsin was deactivated from the supernatants by treatment with 200µg/ml trypsin inhibitor (Sigma Aldrich) for 15m at room temperature. Vero cells were maintained and prepared as described above and treated with a 4-fold dilution series of trypsinised toxin supernatant. Following overnight incubation, the cell monolayer was assessed for cell rounding under an inverted microscope. The number of rounded cells was determined from images taken for each replicate of every strain, and control condition, following incubation with the neat undiluted supernatants.

Western blot detection of CdtA

Supernatants were collected and processed at 48 and 96h as described above. Supernatants (4ml) were mixed with trichloroacetic acid to a final concentration of 10% v/v for 30m on ice before centrifuging at 4°C for 30m at 5049*g*. The resultant protein pellets were washed in 100% ethanol and acetone before suspending in 50µl of PBS and 50µl of 4X NuPage LDS sample buffer (Invitrogen) containing 0.4M dithiothreitol resulting in a 40X protein concentration. Proteins were simultaneously dissolved and denatured in a dry bath at 95°C for 10m before being electrophoresed on a NuPage pre-cast Bis-tris SDS PAGE gel (Invitrogen) for 90m at 100V, after which, proteins were transferred onto a PVDF membrane using Trans-Blot Turbo apparatus (Biorad). Then membranes were blocked for 1h in 30ml of tris-buffered saline (TBS) containing 5% w/v skimmed-milk powder, followed by overnight incubation at 4°C in TBS containing 5% w/v skimmed-milk powder and a 1:1000 dilution of HRP-Chicken anti-*Clostridium difficile* Binary Toxin Subunit A antibody (Gallus-Immunotech). Membranes were washed in 30ml of TBS containing 0.1% Tween 20 (TBST) for 15m before replacing with fresh TBST for a total of 4 washes. Washed membranes were soaked in 5-10ml of 3,3′,5,5′-Tetramethylbenzidine substrate solution (Sigma Aldrich) and developed for 15m prior to scanning and imaging.

**References**

1. Cartman, S.T., et al., *Precise manipulation of the Clostridium difficile chromosome reveals a lack of association between the tcdC genotype and toxin production.* Appl Environ Microbiol, 2012. **78**(13): p. 4683-90.

2. Ng, Y.K., et al., *Expanding the Repertoire of Gene Tools for Precise Manipulation of the <italic>Clostridium difficile</italic> Genome: Allelic Exchange Using <italic>pyrE</italic> Alleles.* PLoS ONE, 2013. **8**(2): p. e56051.

3. Cartman, S.T. and N.P. Minton, *A mariner-Based Transposon System for In Vivo Random Mutagenesis of Clostridium difficile.* Applied and Environmental Microbiology, 2010. **76**(4): p. 1103-1109.

4. Heap, J.T., et al., *Integration of DNA into bacterial chromosomes from plasmids without a counter-selection marker.* Nucleic Acids Res, 2012. **40**(8): p. e59.

5. Williams, D.R., D.I. Young, and M. Young, *Conjugative plasmid transfer from Escherichia coli to Clostridium acetobutylicum.* J Gen Microbiol, 1990. **136**(5): p. 819-26.


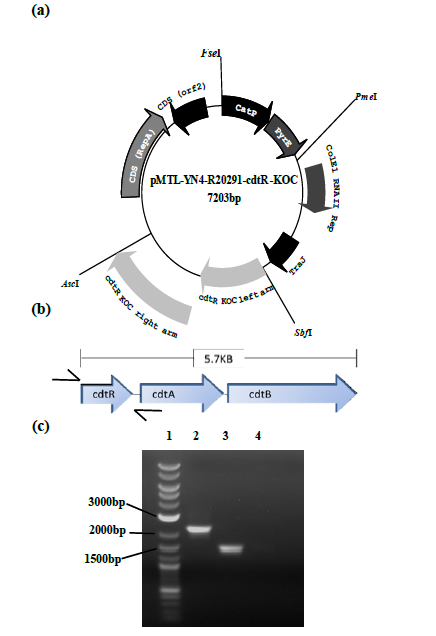


Figure S1: (a) Plasmid map of the Knockout cassette used for the *cdtR* deletions based on pMTL-YN4. (b) Schematic of the wild-type R20291 CDT-Loc displaying the primer binding sites for the cdtR diagnostic forward and reverse primers. (c) Gel image of the cdtR diagnostic PCR using cdtR diagnostic forward and reverse primers (cdtR diag F/R). Lane 1: NEB 2-Log ladder; lane 2: wild-type R20291Δ*pyrE;* lane 3: R20291Δ*pyrE*Δ*cdtR* deletion mutant; lane 4: no-template negative control.


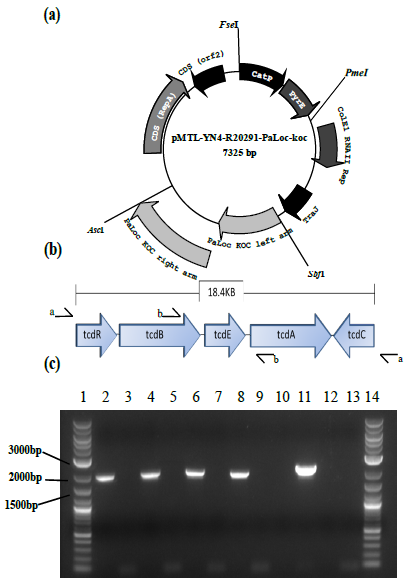


Figure S2: (a) Plasmid map of the Knockout cassette used for the PaLoc deletions based on pMTL-YN4. (b) Schematic of the wild-type PaLoc in R20291 with primer binding sites for the ((a)) PaLoc diagnostic forward and reverse primers (PaLoc diag F/R), and the ((b)) PaLoc internal forward and reverse primers (PaLoc int F/R). (c) Gel image following PCR of the model strains using PaLoc diagnostic and internal primers. Lane 1+14: NEB 2 Log ladder; lane 2-3: R20291∆*pyrE*∆PaLoc clone 1 diagnostic and internal; lane 4-5: R20291∆*pyrE*∆PaLoc clone 2 diagnostic and internal; lane 6-7: R20291∆*pyrE*∆*cdtR*∆PaLoc clone 1 diagnostic and internal; lane 8-9: R20291∆*pyrE*∆*cdtR*∆PaLoc clone 2 diagnostic and internal lane 10-11: wild-type R20291∆*pyrE* diagnostic and internal; lane 12-13: no-template control diagnostic and internal.


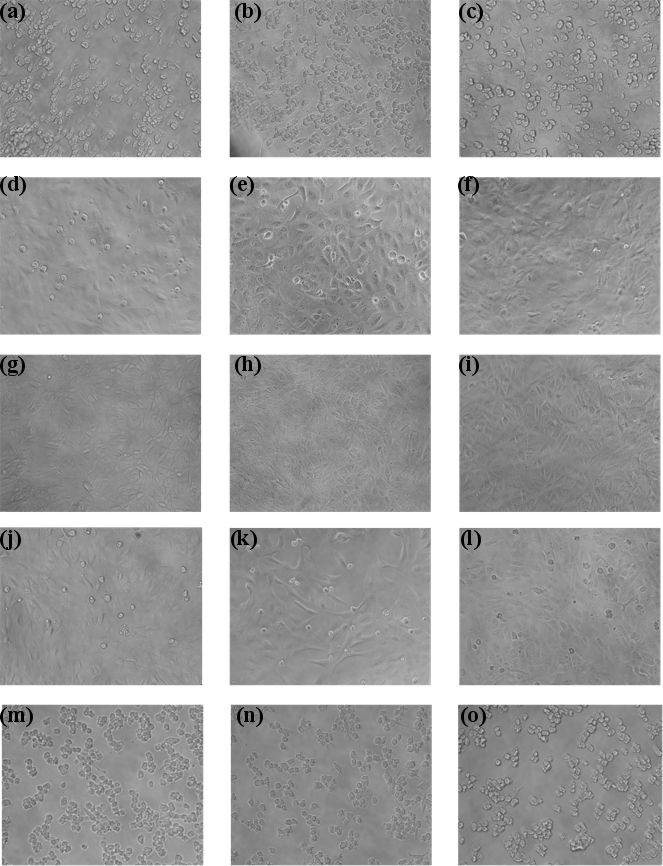


Figure S3: Vero cell-cytotoxicity assay for CDT. Representative images of Vero cells treated with model strain derived supernatants and appropriate controls, (a-c): R20291∆PaLoc; (d-f): R20291∆PaLoc∆*cdtR*; (g-i): trypsin/trypsin inhibitor PBS control; (j-l): R20291∆PaLoc CDT-minus control; (m-o): R20291∆PaLoc∆*cdtR***cdtR*.


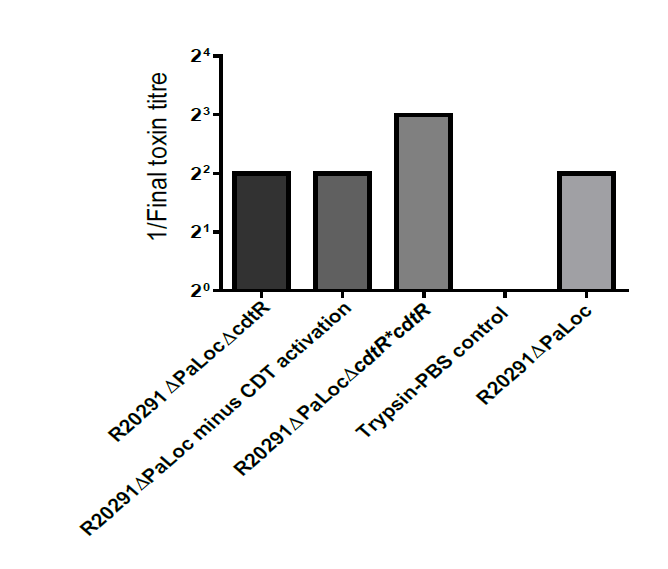


Figure S4: Cytotoxicity assays for CDT using Vero cells treated with 96h supernatants from the relative ΔPaLoc model strains and appropriate controls. The end point represents the dilution at which the cells were comparable to the PBS control. Data represent the mean ±SD of 5 replicate values.

**Table S1: bacterial strains and plasmids**

| Strain/Plasmid | Description | | Reference |
| --- | --- | --- | --- |
| *E. coli*  Top10 | Cloning host. | | Invitrogen |
| CA434 | Conjugal donor. | | [[5](#_ENREF_5)] |
| *C. difficile*  R20291 | Clinical RT 027 isolate | | J. Brazier, Anaerobe Reference  Laboratory, Cardiff, United  Kingdom |
| R20291∆*pyrE* | Mutant for allelic exchange manipulation. | | [[2](#_ENREF_2)] |
| R20291∆*pyrE*∆*cdtR* | Initial mutant | | This study |
| R20291∆*pyrE*∆PaLoc | Initial mutant | | This study |
| R20291∆*pyrE*∆PaLoc∆*cdtR* | Initial mutant | | This study |
| R20291∆*cdtR* | *pyrE* restored mutant. | | This study |
| R20291∆PaLoc | *pyrE* restored mutant. | | This study |
| R20291∆PaLoc∆*cdtR* | *pyrE* restored mutant. | | This study |
| R20291∆PaLoc∆*cdtR***cdtR*.  Plasmid  pMTL-YN4  pMTL-YN4-*cdtR* KOC  pMTL-YN4-PaLoc KOC  pMTL-YN2  pMTL-YN2C  pMTL-YN2C-*cdtR* | cdtR complemented at *pyrE* locus  Knockout vector for R20291  As above with *cdtR* deletion insert  As above with PaLoc insert  Restoration of *pyrE*  Restoration of *pyrE* and insertion of complemented sequence  As above with *cdtR* insert | | This study  [[2](#_ENREF_2)]  Fig. S1(a)  Fig. S2(a)  [[2](#_ENREF_2)]  [[2](#_ENREF_2)]  This study |
|  |  | |  |
|  |  | |  |
|  |  |  |  |
|  |  | |  |

**Table S2: oligonucleotide primers**

| Primer pair | Sequence 5’-3’ | |  |
| --- | --- | --- | --- |
| *cdtR* Knockout |  | |  |
| *cdtR* KOC LAF | TTTTTTCCTGCAGGTAATAACTTGTAGTTATCATTAACTCTACAATTC | |  |
| *cdtR* KOC LAR | TTTCTATTTATAAATACCCTCCTATAAAAAATTC | |  |
| *cdtR* KOC RAF | GGAGGGTATTTATAAATAGAAAAAAGAGATGTCTC | |  |
| *cdtR* KOC RAR | AAAAAAGGCGCGCCTGAGAATATTTACTTATTTCTACAGAATCTTTTT | |  |
| *cdtR* diag F | ACTTCTATTAACAACTAATACTAGCTGACTAACAC | |  |
| *cdtR* diag R | TTTCTCGAGAATTTGCTTCTATTTGATAATCATAA | |  |
|  |  | |  |
| PaLoc Knockout |  | |  |
| PaLoc KOC LAF | TTTTTCCTGCAGGCGAAGAGGAGCTAACAGAG | |  |
| PaLoc KOC LAR | TATTTTGGTGGACAACATTGGAATTAAATCAG | |  |
| PaLoc KOC RAF | CAATGTTGTCCACCAAAATAAATGCCAGTAG | |  |
| PaLoc KOC RAR | TTTTTGGCGCGCCGATGGGTATATTTAGCCATAC | |  |
| PaLoc diag F | GGCAAGTGTATGTATTATAC | |  |
| PaLoc diag R | CCTTTGTTAGGTAGTATGTTTAG | |  |
| PaLoc int F | GATAATATCTATGGACAAGCAGTTG | |  |
| PaLoc int R | CAATATCACTGACTTCTCCACC | |  |
| Single cross-over (SCO) determination |  | |  |
| YN4 F | CTCCATCAAGAAGAGCGAC | |  |
| YN4 R | CTTATCCAGGGTGCTATC | |  |
| *pyrE* restoration |  | |  |
| pyrE WT F | GGAGCTACTTGTATCCAAG | |  |
| pyrE WT R | CCTAATTCCTTGAACTCTC | |  |
| pyrE LacZ R | CGTGACGTCGACTCTAG | |  |
|  |  | |  |
| *cdtR* complementation |  | |  |
| Promoter-cdtR F | TTTTTGCGGCCGCCACGTATAAGAATAAAAATTCCAG | |  |
| *cdtR* R | TTTTTGGATCCTTATGTTTTAATAATGTTCTTTAAAATATTTC | |  |
|  |  |  | |
